# Nurses’ perceptions of caring activities in nursing

**DOI:** 10.1002/nop2.653

**Published:** 2020-10-10

**Authors:** Neriman Akansel, Roger Watson, Nursel Vatansever, Aysel Özdemir

**Affiliations:** ^1^ Department of Nursing Bursa Uludag University Faculty of Health Sciences Bursa Turkey; ^2^ FAAN Professor of Nursing University of Hull Faculty of Health and Social Care Hull UK

**Keywords:** caring, item response theory, Mokken scaling, nursing, Turkey

## Abstract

**Aim:**

This study aimed to determine nurses’ perceptions of caring activities in nursing.

**Design:**

A descriptive study design.

**Methods:**

A Turkish translation of the 25‐item version of the Caring Dimensions Inventory was completed by 260 nurses working in one university hospital. Data were analysed using Mokken scaling.

**Results:**

Technical aspects of nursing were highly endorsed items such as “observing the effects of a medication on a patient, measuring vital signs, being technically competent with a clinical procedure, consulting with the doctor” except for the item “providing privacy for a patient” which is a psychosocial item. The range of items included in the Mokken scale with “providing privacy for a patient” (mean = 4.31) as the most endorsed, and “exploring the patient's lifestyle” (mean = 2.60) being the least endorsed item. Listening to patients and involving them in their care are not considered as caring.

## 1 INTRODUCTION

Nurses are unique caregivers that make a difference in patients’ lives. Nurses aim to protect, promote and optimize the health of individuals, preventing illness, facilitating healing, alleviate suffering through the diagnostic procedures and advocate in the care of individuals and families (American Nurses Association, https://www.nursingworld.org/practice-policy/scope-of-practice/; accessed 10 June 2020). Finkelman and Kenner (2013) underlines that caring relationship, attention to human responses, integrating assessment data, application of the scientific data, advancing professional nursing knowledge, promoting social justice, assuring safe and evidence‐based practice are features of professional nursing. Professional, personal, scientific, aesthetic and ethical human transactions are important in nursing where a patient should be a focus of practice (Kandula, 2019).

## 2 BACKGROUND

Caring in the nursing profession is a challenging, universal phenomenon yet a difficult process for nurses to understand and articulate. Nevertheless, theorists agree that caring is an essential aspect of the nursing profession (Alpar et al., 2013), and the value of nursing care on positive outcomes in patients’ well‐being is undeniable(Ayyub et al., 2015). Nevertheless, nurse caring is considered as a quality indicator in healthcare organizations (Burtson & Stichler, 2010). As it launched in Watson's Caring Theory, caring occurs whenever a nurse and patient come in to contact with each other's. The theory emphasizes the interactions between the caregiver and recipient; where holistic nursing care is placed in the centre of caring (Kandula, 2019). Holistic nursing and nursing care which is in harmony with the culture are vital components of Leininger's theory of Transcultural Nursing as well (Alpar et al., 2013).

The magnitude of caring in nursing is very complex (Finkelman & Kenner, 2013); thus, studies conducted on caring in nursing reveal different dimensions of caring as well as different descriptions. Nursing requires a range of technical and psychological dimensions and has other dimensions that are not clarified (Lea et al., 1998). Finkelman and Kenner (2013) emphasizes that practising as a nurse is far beyond the basic knowledge on how to do specific things or the ability to care for someone. Being able to care for someone requires attentiveness, concern and knowledge and art of caring (Finkelman & Kenner, 2013; Hudacek, 2008b).

The latest definition is caring provided by the American Association of the Critical Nurses’ Synergy Model for Patient Care is: “activities performed by nurses in a compassionate, supportive and therapeutic environment to promote comfort and healing. Nursing care also should focus on preventing unnecessary suffering as well.” (American Association of Critical Care Nurses, https://www.aacn.org/nursing-excellence/aacn-standards/synergy-model,2017; accessed 10 June 2020). According to this description, caring includes both attitudes and activities (actions) performed by nurses. Research shows that nurses’ caring behaviours are influenced by several factors such as working conditions; workload; management support; and concerns related to patients’ health (Enss & Swatzky Jo‐Ann, 2016), compassion fatigue (Burtson & Stichler, 2010), cultural differences (Ian et al., 2016; Watson, 2003), patients’ expectations from nurses (Özbaşaran et al., 2000; Weyant et al., 2017) and nurses’ perceptions about caring (Karaöz, 2005; Skott & Erikson, 2005).

Hudacek (2008a) says the personal meaning of caring for nurses should be questioned. In addition to personal perceptions of caring, compassion, spirituality, being aware of people who need health care, assessing patients' comfort, providing comfort, crisis intervention, undertaking advocacy roles both for the patient and their families contribute value to the nursing profession should not be underestimated (Hudacek, 2008b).

Rego et al. (2010) indicate that high empathy levels of nurses provoke an increase in their caring behaviours. Some studies emphasize that altruistic and emotional aspects (O’Connell & Lenders, 2008), intimacy and support aspects (Watson et al., 2001), usually comes after technical aspects of nursing according to nurses and nursing students. Despite the fact that nurses are taught how to care properly for patients during their education; their practice and perceptions related to caring are quite different. Watson et al. (2001) report that other aspects of nursing such as intimacy and support develop sometime later in nursing students during their education. Another study suggests that although professional values tend to increase during nursing education compassion of students does not differ greatly (Kavradım et al., 2019). Thus, clinical practice plays a significant role in developing caring behaviours in nursing students (Mlinar et al., 2010). Therefore, using narratives is recommended to use in nursing education to expand awareness of nursing students on caring (Hudacek, 2008a). Overall, whenever the person can meet his/her caring needs in daily life, he/she tends to show sensitivity to the caring needs of others (Baykara & Şahinoğlu, 2014; Öztunç, 2013). One study involving nursing students emphasizes that students who willingly choose nursing as a career and the ones’ who had caring experience are more sensitive to patient needs (Birimoğlu & Ayaz, 2015). Caring perceptions of nursing students are influenced by their attitudes and experiences (Konuk & Tanyer, 2019). There is a need to support nursing students during their education using appropriate methods of teaching and role models are essential (Culha & Acaroğlu, 2019). Overall, nursing students should be taught how to care for themselves at first because being able to take care of the others is consuming a lot of energy and draining experience as well. This is also a very important practice for qualified nurses since nurses tend to ascribe different meanings to caring in nursing.

Although most of the studies involving nurses and patients show that technical aspects of nursing are an important part of their perceptions related to caring (Acaroğlu et al., 2009; Algıer et al., 2005; Ayyub et al., 2015; Geçkil et al., 2008; O’Connell & Lenders, 2008; Özdemir & Şenol Çelik, 2010), psychological dimensions of nursing care should not be underestimated (Ayyub et al., 2015), as well as providing reassurance to patients (Weyant et al., 2017) being his/her advocate (Hudacek, 2008a) and providing culturally appropriate care (Murphy et al., 2009). Area of practice (e.g. surgical and medical) also influences nurses caring behaviours (Lea & Watson, 1999; Walsh, 1999; Watson & Lea, 1998). Nurses who are exposed to occupational stress tend to have a low quality of life which can also influence patient outcomes (Sarafis et al., 2016). ICU nurses report that working with dying patients is a stressful, draining, depressing and heartbreaking experience. Also, supporting families is another dimension of care for ICU nurses (Kisorio & Langley, 2016). According to critical care nurses and the relatives of critically ill patients, technical aspects of nursing are important (O’Connell & Lenders, 2008), this aspect of nursing is also valuable for nursing students as reported by different studies (Akansel et al., 2012 and Watson et al., 2001). Researchers report that gender has an influence on which caring behaviours were valued among nurses the most (Greenhalgh et al., 1998; Lea & Watson, 1999; Watson & Lea, 1998).

Differences were also found between nurses’ and patients’ perceptions about caring (Geçkil et al., 2008). Nurses should be familiar with patients’ perceptions of caring and instill this in improving their nursing care (McCance et al., 2008). Patients have expressed dissatisfaction with how nurses react to their worries and fears, providing comfort during hospitalization (Geçkil et al., 2008). Most of the studies on this topic reveal different perceptions of caring by patients and nurses. McCance et al. (2008) reveal that both technical and intimacy dimensions of nursing were prioritized by nurses. Specifically, “listening to a patient” underlined as a part of nursing care by nurses. In the same study, patients emphasized “involving a patient in care” and “providing privacy for a patient” as nursing care. Consequently, perceptions of nurses and patients’ on caring are incongruent. The respectful and holistic approach provided by advanced practice nurses in Sweden combined with knowledge and skills is stressed as an important part of caring by patients (Eriksson et al., 2018). A systematic review by Papastavrou et al. (2011) emphasized that instrumental nursing skills and nurses' competency in those skills are important for patients. However, effective nursing care is perceived as expressive care for nurses which differs from patients’ perspective. Visible features of nursing care are valuable to patients while invisible caring activities such as expounding the nursing care, protecting the patient and competency are underlined by nurses (Canzan et al., 2014).

Since nurses' perceptions of caring are influenced by numerous facts, it is important to clarify the nurses' understanding of the phenomenon of caring. As far as we know of no study that determines the caring dimension of the working nurses using the Caring Dimensions Inventory Turkey (CDI‐25).

### 2.1 Aim

This study aimed to determine nurses’ perceptions about caring activities in nursing by using CDI‐25 and compare the findings with relevant literature.

## 3 METHODS

### 3.1 Design

Demographic variables and data on nurses working status were collected using a data collection form which consisted of 10 questions.

‐Four questions were related to demographic variables of nurses.

‐Five questions were related to nurses working experience, number of patients assigned, wards they work.

‐One question used to determine nurses’ perception of the efficiency of nursing care given to patients using the Visual Analogue Scale (0 = not efficient, 10 = completely efficient).

The Turkish translation of the CDI‐25 (Akansel et al., 2012) self‐administered questionnaire for measuring nurses’ perceptions about caring originally developed by Watson and Lea (1997) was used for data collection. CDI‐25 includes twenty‐five statements of nursing actions. In the study of Lea et al. (1998), CDI‐25 was categorized into five dimensions; psychosocial, technical, professional, inappropriate and unnecessary activities. There is a base question in the inventory: “Do you consider the following aspects of your nursing practice to be caring?” CDI‐25 includes statements of nursing actions. Respondents answer the items included in the inventory through a 1–5 point scale (1 = disagree and 5 strongly agree). Chronbach's alpha value of CDI‐25 was calculated as 0.91 indicating that it had a high degree of internal consistency (Watson & Lea, 1997). In the Turkish form of the instrument, the ordering of participants was supported by appropriate Mokken Scaling Parameters and scoring of items by participants was not invariant. The Turkish version of the Caring Dimensions Inventory is a reliable instrument for measuring nurses’ perceptions about caring. Mostly, endorsed items were psychosocial while the professional/technical items were less endorsed (Akansel et al., 2012).

### 3.2 Sample and data collection procedure

Data were collected during September 2015‐February 2016 by two of the researchers in one university hospital in the northwest region of Turkey. Four hundred fifty (*N* = 450) nurses were employed in the hospital during the conduction of this study. Operating room nurses and outpatient clinic nurses were excluded from the study since uninterrupted patient care is not available in those departments. Nurses who were on sick leave, annual leave or unpaid leave were also excluded making. Data were collected from 280 nurses who volunteered to participate in this study. Since 20 participants did not complete the forms properly (missing data on the data collection form and CDI‐25 inventory), study was completed with 260 nurses Researchers handed the forms (data collection form and CDI‐25 inventory) in envelopes to the nurses and collected completed forms after one week.

### 3.3 Mokken scaling

The present study used the non‐parametric item response (IRT) theory method of Mokken scaling analysis (MSA). IRT methods offer advantages over the more commonly applied multivariate methods based on such as factor analysis—based on classical test theory—in that they can establish item ordering in scales (hierarchies) and, thereby, provide a more meaningful relationship between scale scores and levels of the latent trait being investigated. A non‐technical explanation of MSA has been provided by Watson et al. (2012) where the underlying principles of the method are explained along with the parameters whereby the qualities of a Mokken scale may be judged. These parameters include Loevinger's coefficient (H) which is a measure of the strength of an overall scale (Hs) or the scalability of individual items (Hi) and pairs of items (Hij). The minimum requirement for values of H is that they equal or exceed 0.30; the lower‐bound 95% confidence intervals for individual items should not include 0.30 and the lower‐bound 95% confidence intervals for item pairs should not include 0 (Kuijpers et al., 2013). Values of Hs equalling or exceeding 0.30, 0.40 or 0.50 indicate weak, moderate and strong scales, respectively. Items scores should continually increase as the latent trait increases (monotonicity) and items characteristic curves (ICC)—which describe the relationship between the score on an item and the level of the latent trait—should not intersect, a property knows as invariant item ordering (IIO). Monotonicity is judged using a “Crit” value, which is calculated from the number of violations of monotonicity; values should not exceed 80 (Molenaar & Sijtsma, 2000). The existence of IIO can be established by a combination of visually inspecting plots of ICCs and looking for significant violations of IIO; the strength of IIO can be estimated using Htrans (H^T^) (Watson et al., 2014) which is analogous to Hs and the values for judging the strength of IIO are the same as those for Hs reported above. The probability of obtaining a Mokken scale and the reliability (rho) of Mokken scales can also be estimated.

### 3.4 Data analysis

Data were entered into SPSS 21.0 and checked listwise for missing values before conversion in R (public domain software available at https://www.r-project.org/; accessed 18 August 2015) using the “foreign” package to a form suitable for analysis in R using the “mokken” package. Data were analysed using the automated item selection procedure (“aisp” which has a default setting of Hs 0.30, *P* < .05 and which finds the item with the highest Hi and then builds a Mokken scale until no further items fit) and inspected for violations of monotonicity. Standard errors of Hs and Hij were used to check 95% confidence intervals. Item pairs were plotted and inspected for intersection and for any outlying items which may exaggerate IIO. The data were then analysed for significant violations of IIO and items sequentially removed until no further violations were evident.

### 3.5 Ethics

Ethical approval was given by the University Ethical Board (2014‐2015/10. B.30.2.ULU.0.20.70.02.050.99/290) for this study. Permission was obtained from the author to use the scale (CDI‐25 Turkish Version) in this study. All of the nurses were informed about the aim of the study both verbally and in writing. They were reassured that participation is voluntary and have the right to leave the research at any time they want to.

## 4 RESULTS

The CDI was administered to 280 nurses and 260 completed it giving a response rate of 92.8%. Nurses’ ages ranged between 20–53 years (mean = 32.34, *SD* 6.14). As shown in Table 1 most of the nurses were female (93.8%) and had a bachelor's degree in nursing (78.8%). The mean working experience of nurses was 9.76 (*SD* 6.46) years. According to nurses working in different wards of the hospital, the efficiency of care given to hospitalized patients was 7.14 (*SD* 1.83) according to the Visual Analogue Scale numbered 0–10. Data from two participants were removed due to missing data, and 18 nurses did not want to participate.

**TABLE 1:** Demographic variables of working nurses (*n* = 260)

	Mean ± *SD* (range)
Age	32.3 ± 6.1 (range 20–53 years)
Work experince as a nurse (years)	9.8 ± 6.5 (range 1–31 years)
Mean number of patients asiigned per nurse (in one shift)	10.5 ± 7.6 (range 1–40)
Nurses’ perception on effciencey of nursing care given to patients	7.1 ± 1.8 (range 1–10)
	Numbers (percentage %)
Gender	
Female	244 (93.8%)
Male	16 (6.2%)
Marital status	
Married	177 (68.1%)
Single	83 (31.9%)
Degree	
High school degree	12 (4.6%)
Associate degree	20 (7.7%)
Bachelor degree	205 (78.8%)
Msc in Nursing	23 (8.8%)
Position	
Staff nurse	246 (94.6%)
Head nurse	14 (5.4%)
Ward	
Medical Unit	72 (27.7%)
Surgical Unit	81 (31.2%)
ICU	90 (34.6%)
ER	12 (4.6%)
Pediatric Clinic	5 (1.9%)
Working shifts of nurses	
Rotating shifts (08−16/ 16–08)	185 (71.2%)
Always 08–16 shifts	49 (18.8%)
Always 16–08 shifts	26 (10.0%)

The analysis showed a single scale with four items (8, 16, 17 & 19) not scaling which means that these items did not meet the basic scalability requirement of having Hi ≥ 0.3 and were therefore excluded from the analysis. Items 3, 7, 8, 17 and 18 had lower bound 95% confidence intervals including 0.30; this means that 95% of the time these items are included in a Mokken scaling analysis they will not meet the minimum requirement of Hi ≥ 0.3. Nevertheless, they were not removed from the analysis as the confidence interval may be related to the relatively small sample size. All item pairs with item 16 had 95% confidence intervals including 0, and this is a clear indication that there may be a problem with item 16 in relation to the other items in the scale. This was verified by visual inspection of item pair plots which showed that the ICC for item 16 was positioned a long way from the remaining cluster of items; therefore, item 16 was removed from the analysis of IIO.

Table 2 shows the outcome of sequentially removing items until there were no further violations of IIO—in other words that there were no overlapping items—leaving 19 items in 6 steps with a final H^T ^of 0.40 indicating moderately strong IIO whereby items are reasonably positioned along the span of the latent trait.

**TABLE 2:** Number of significant violations of IIO and effect on Htrans (*H^T^*) with sequential item removal steps (*N* = 260)

Item	Label	Step 1	Step 2	Step 3	Step 4	Step 5	Step 6
1	Assisting a patient with an activity of daily living (washing, dressing, etc.	0	0	0	0	0	0
2	Making a nursing record about the patient	0	0	2	–	–	–
3	Feeling sorry for a patient	0	1	0	1	–	–
4	Getting to know the patient as a person	1	0	0	0	0	0
5	Explaining a clinical procedure to a patient	0	0	0	0	0	0
6	Being neatly dressed when working with a patient	0	0	0	0	0	0
7	Sitting with a patient	0	0	0	0	0	0
8	Exploring a patient's lifestyle	0	0	0	0	0	0
9	Reporting a patient's condition to a senior nurse	0	0	0	1	0	0
10	Being with a patient during a clinical procedure	1	0	0	0	0	0
11	Being honest with a patient	2	–	–	–	–	–
12	Organizing the work of others for a patient	0	0	1	0	0	0
13	Listening to a patient	0	0	0	0	0	0
14	Consulting with a doctor about a patient	0	0	0	0	0	0
15	Instructing a patient about an aspect of self‐care (washing, dressing, etc.)	1	0	1	1	1	0
16	Sharing your personal problems with a patient*	–	–	–	–	–	–
17	Keeping relatives informed about a patient	0	0	0	0	0	0
18	Measuring the vital signs of a patient (e.g. pulse and blood pressure)	0	0	0	0	0	0
19	Putting the needs of a patient before your own	0	1	–	–	–	–
20	Being technically competent with a clinical procedure	0	0	1	0	0	0
21	Involving a patient with his or her care	0	0	0	0	0	0
22	Giving reassurance about a clinical procedure	0	0	0	1	0	0
23	Providing privacy for a patient	0	0	0	0	0	0
24	Being cheerful with a patient	1	0	1	1	1	–
25	Observing the effects of a medication on a patient	0	0	0	0	0	0
*Removed following inspection of item pair plots	*H^T^*	0.35	0.35	0.36	0.38	0.39	0.40

Table 3 shows all of the items ordered by the mean score with those showing IIO and those with unsatisfactory 95% confidence intervals indicated. The most highly endorsed item is “Providing privacy for a patient” and the least endorsed is “Exploring a patient's lifestyle.” However, it should be noted that three of the items showing IIO had 95% confidence intervals which included 0.30.

**TABLE 3:** Mokken scale of CDI‐25 items (*N* = 260)

Item	Label	Mean score	*Hi(SE)*
23	Providing privacy for a patient	4.31	0.50 (0.03)*
25	Observing the effects of a medication on a patient	4.21	0.51 (0.03)*
18	Measuring the vital signs of a patient (e.g. pulse and blood pressure)	4.16	0.47 (0.04)*
20	Being technically competent with a clinical procedure	4.10	0.46(0.04)*
14	Consulting with a doctor about a patient	3.99	0.49 (0.03)*
15	Instructing a patient about an aspect of self‐care (washing, dressing, etc.)	3.90	0.48 (0.03)*
24	Being cheerful with a patient	3.89	0.45 (0.04)
13	Listening to a patient	3.85	0.52 (0.04)*
21	Involving a patient with his or her care	3.75	0.40 (0.04)*
22	Giving reassurance about a clinical procedure	3.73	0.39 (0.04)*
12	Organizing the work of others for a patient	3.71	0.46 (0.04)*
5	Explaining a clinical procedure to a patient	3.71 0.49 (0.03)*	
2	Making a nursing record about the patient	3.70	0.40 (0.04)
4	Getting to know the patient as a person	3.66	0.44 (0.03)*
11	Being honest with a patient	3.66	0.44 (0.04)
10	Being with a patient during a clinical procedure	3.57	0.50 (0.03)*
6	Being neatly dressed when working with a patient	3.54	0.44 (0.04)*
1	Assisting a patient with an activity of daily living (washing, dressing, etc.	3.43	0.40 (0.04)*
9	Reporting a patient's condition to a senior nurse	3.10	0.41 (0.03)*
3	Feeling sorry for a patient	3.05	0.30 (0.04)^†^
19	Putting the needs of a patient before your own	2.95	0.30 (0.04)^†^
17	Keeping relatives informed about a patient	2.94	0.24 (0.05)*^†^
7	Sitting with a patient	2.69	0.35 (0.04)*^†^
8	Exploring a patient's lifestyle	2.60	0.26 (0.05)*^†^
16	Sharing your personal problems with a patient	1.36	−0.16 (0.09)

Note

Hs = 0.40; Rho = 0.93; * = items showing IIO (*H^T^* = 0.40); † = items with lower 95% confidence intervals <0.30.

## 5 DISCUSSION

### 5.1 Concepts of Caring in Nursing

Nursing care is composed of professional understanding, knowledge and skills to practice nursing and interactions between nurses and patients (Dinç, 2010). According to Leininger's theory of Transcultural Nursing, nursing is described as a science and type of art which can be learned. Caring is an essence of nursing and basic human need. Every single culture has some differences and similarities in terms of ethical and moral values. Thus, nurses are expected to give care to their patients in a holistic manner, suitable for their cultural structure and also be respectful to it (Alpar et al., 2013).

Being able to give sufficient, professional and culturally sensitive care to an individual is an important aspect of nursing (Murphy et al., 2009). While teaching these aspects of caring in nursing schools, there is a great emphasis on how to provide patient care properly. Thus, both teaching and learning nursing is a process that requires effort both by nursing faculties and nursing students. Both the definition and provision of caring activities in nursing are difficult and multidimensional. There is no universally accepted definition of the concept of caring. Caring includes perspectives such as compassion, or being concerned about another person; doing for other people what they cannot do for themselves; care for the medical problem and competence in carrying. Nurses conduct caring activities daily. However not every aspect of caring needs to be used by nurses at one time for nursing care to be effective (Finkelman & Kenner, 2013).

### 5.2 Perceptions of Caring Among Nurses

A perception of caring in nursing has different meanings for nurses, nursing students, patients and their families, and this perception has been shaped by several factors. Concurrently, nurses’ and patients’ perceptions related to caring are incongruent (Papastavrou et al., 2011). Studies that focus on perceptions of nurses about caring are very few in Turkey (Acaroğlu et al., 2009; Karadağ & Taşçı, 2005; Kılıç & Öztunç, 2015). Most nurses emphasize the psychological aspects of nursing as important in caring but they usually rely on technical activities because of reasons such as the limited number of staff, long working hours and high patient ratios. Kılıç and Öztunç’s (2008) study reveals relieving patient's pain, giving medications on time as prioritized by nursing staff; both patients and nurses’ privileged “being with a patient” as an important nursing care activity. A study by O’Connell and Landers (2008) shows that ICU nurses and relatives of the patients highly value technical competence and the altruistic and emotional aspects of nursing as a caring behaviour. Patients usu**a**lly focus on nurses’ technical skills as well as their competency (Geçkil et al., 2008; Wysong & Driver, 2009), since they believe good technical skills reduce their discomfort and lead to less pain (Ayyub et al., 2015). In this study, aspects of care such as observing the effects of being technically competent with a clinical procedure, consulting with the doctor, instructing a patient about aspects of care, listening to a patient, explaining the clinical procedure to a patient and being with a patient during the clinical procedure were among the endorsed items which are congruent with two previous studies (Akansel et al., 2012; Watson et al., 2003) although the order of the items is different. Items referring to technical aspects of nursing were found to be among the highly endorsed items. Observing the effects of a medication on a patient item was included among the second most highly endorsed items in our current study followed by measuring the vital signs, of a patient, being technically competent with the clinical procedure. According to the results of Pajnkihar et al. (2019), Slovene and Russian nurses also rated the item related to medication administration highly. While “observing the effects of a medication on a patient” was the less endorsed item in the UK study (Watson et al., 2003); the same item was not endorsed in the previous study done with Turkish nursing students (Akansel et al., 2012) as well as in Persian study done with Iranian nurses (Salimi et al., 2014).

In Turkey because of high patient ratios per nurse in clinical environments, this may lead nurses to think about the importance of “observing the effects of medications” and “consulting the doctor” as a priority. In addition to this, physicians are the ones who are primarily arranging the treatment of the patient, notifying the doctor may have been evaluated as an important aspect of the nursing care in this study. Besides, working on rotating shifts may have influenced the nurses’ perceptions. Night shifts especially exert pressure on nurses because of limited staff and having to make critical decisions in limited time may be worrisome. Concurrently, night shifts and long working hours may also have a destructive effect on nurses. Decision regret is reported to be common among nurses who work more than 12‐hr shifts which link to less sleep and not being able to rest well (Scott et al., 2014). Being unable to make critical decisions is an important problem in inpatient settings that could interfere with patient safety. Different studies report that nurses who work on night shifts are more prone to sleep problems (Books et al., 2017; Ferreira et al., 2017) family stressors and mood changes (Books et al., 2017).

“Taking vital signs” which is another technical aspect of nursing has seen as an important caring activity in our study. Assessment of vital signs and evaluating the results are valuable activities in nursing that could guide the treatment of the patient. This aspect of nursing is considered a time‐consuming activity because of the number of patients assigned to each nurse (mean: 10, 46 *SD* 7, 57) in our study. This result can be interpreted as either because nurses usually able to assess their patients during taking vital signs and evaluate the changes that may have to occur on the patient, or they perceive this activity as primary nursing care in their practice. Being technically competent was found to be in the same order in the study with Turkish nursing students (Akansel et al., 2012), and it is quite different from the results of Spanish and UK nurses (Watson et al., 2003). The results of our study are like those conducted previously where take notice of technical aspects of nursing as caring (Acaroğlu et al., 2009; Akansel et al., 2012; Ayyub et al., 2015; Geçkil et al., 2008; Karadağ & Taşçı, 2005; Karaöz, 2005; Weyant et al., 2017).

According to these studies, it can be considered that priorities in nursing care activities could change according to patients’ and nurses’ perspectives and expectations. Also, social, cultural and religious beliefs, the caring culture of the facilities, individual variations and willingness to practice the nursing profession may influence caring perceptions of nurses. Geçkil et al. (2008) emphasized that the protection of privacy by nurses afforded the most satisfaction to patients from the nursing care they receive. In this study “providing privacy for a patient” was the most endorsed item which is the same as a previous study with Turkish nursing students (Akansel et al., 2012). This result could be linked to the cultural structure and the beliefs of Turkish people where privacy takes an important place. Providing privacy was the second most endorsed item in UK nurses and the fourth item in Spanish nurses (Watson et al., 2003) (Table 4).

**TABLE 4:** Items common to Mokken scales from UK nurses, Turkish nursing students and Turkish nurses

Mokken scale derived from UK nurses CDI−25 data	Mokken scale derived from Turkish nursing students CDI−25 data	Mokken scale derived from Turkish nurses CDI−25 data
Measuring the vital signs of the patient Consulting with the doctor about a patient* Reporting the patients' condition to a senior nurse Being technically competent with a clinical procedur**e** * Instructing a patient about an aspect of self‐care* Observing the effects of medication on a patient Explaining a clinical procedure to a patient* Being with a patient during a clinical procedure * Involving the patient with his or her care Giving reassurance about a clinical procedure Providing privacy for a patient* Listening to a patient*	Being with a patient during a clinical procedure * Explaining a clinical procedure to a patient* Consulting with the doctor about a patient * Instructing a patient about an aspect of self‐care * Being technically competent with a clinical procedure * Listening to a patient* Being cheerful with a patient Providing privacy for a patient*	Reporting a patient's condition to a senior nurse Assisting a patient with an activity of daily living (washing, dressing, etc.) Being neatly dressed when working with a patient Being with a patient during a clinical procedure* Being honest with a patient Getting to know the patient as a person Making a nursing record about the patient Explaining a clinical procedure to a patient* Organizing the work of others for a patient Giving reassurance about a clinical procedure Involving a patient with his or her care Listening to a patient* Being cheerful with a patient Instructing a patient about an aspect of self‐care (washing, dressing, etc)* Consulting with a doctor about a patient* Being technically competent with a clinical procedure* Measuring the vital signs of a patient (e.g., pulse and blood pressure) Observing the effects of a medication on a patient Providing privacy for a patient*

* Items common to mokken scales from the UK, Turkish nursing students and Turkish nurses (All of the items were listed according to their mean scores.).

Nurses caring behaviours have a great influence on patients’ satisfaction (Azizi‐Fini et al., 2012). Although patients and relatives emphasize the technical aspects of nursing; the kindness of nurses is a valuable thing that is hard for them to forget easily even after leaving the hospital, touching (Algıer et al., 2005) part of effective nursing care by patients. Having a positive attitude and smiling is worthy for patients in the evaluation of the quality of nursing care (Ayyub et al., 2015; Wysong & Driver, 2009). Patients in critical conditions and treated in ICUs are usually influenced by physical, physiological and environmental stressors (Dedeli & Akyol, 2008). Pain, tubes, not being dressed during their ICU stay, unusual noises and continual lighting are some of the stressors patients usually remember even after being discharged from the hospital. Studies reveal that not having privacy in ICU is considered an important stressor for patients (Aktaş et al., 2015; Zaybak & Çevik, 2015); however, nurses did not give this the same priority as patients (Zaybak & Çevik, 2015). Novaes et al. (1997) reported that lack of privacy is not considered a priority among all stressors especially by male patients in ICU. Emergency room (ER) patients usually experience some kind of privacy infringements according to Karro et al. (2005). This situation causes patients to withhold the information from caregivers which may lead to serious adverse effects on treatment and care of the individual. Outcomes of studies related to stressors perceived by patients could be linked to several factors such as physical environment, patient experiences and cultural disparities (Dedeli & Akyol, 2008). Nurses’ sensibility to this issue could be linked to their cultural and moral values. Spiritual distress occurs when individuals are detached from their culture and religious rituals. Culture is composed of different features such as geography, social life and language. Leininger emphasizes that health is a substantial fact in transcultural nursing; since every single culture has its values, beliefs and practices (Alpar et al., 2013). Ian et al.’s study (2016) suggests that nursing for non‐English speaking patients influences nurses’ productivity and their ability to care for the patients. That is why nurses should be equipped with knowledge of cultural issues and awareness of patient needs. Resources should also be available to help nurses communicate effectively with patients. While patients have a right to be treated according to their cultural values, the necessity of giving culturally sensitive care to patients by nurses, gaining basic knowledge are important facts in nursing.

Providing privacy should not be limited to the protection of body parts of the patient; seeing body parts of others’ is also an embarrassing experience for patients. Not sharing patients’ information with the ones who are not primarily responsible for them should also be considered as protecting the privacy of the patients Protecting patients' privacy and giving adequate information (Kim et al., 2016), providing confidentiality, disseminating databases inpatient care centres should be a part of this practice (Onna‐Machado et al., 2004).

Psychosocial aspects of nursing such as sparing time for the patients are important concepts that should not be overlooked in good nursing care. Studies reveal that being the presence of nurses is a valuable circumstance for hospitalized patients that helps them to overcome their fears and concerns (Mohammadipour et al., 2017; Weyant et al., 2017). Listening to patients and involving them in their care should be considered as important caring activities in nursing. However, in this study, these items were not included among highly endorsed items. Listening to a patient was among highly endorsed items in previous studies done using CDI‐25 inventory. However, the results of this study show that this item endorsed even after the ordering found in a study done with Turkish nursing students who describe the psychosocial aspect of nursing care. This result could also be interpreted as that nurses are not able to spare time to getting to know and listening to the patient because of high patient ratios. Most of our study population consisted of nurses who work in medical, surgical units and ICUs. The complexity of the patients being cared for in these units may have influenced the nurses’ perceptions while prioritizing the nursing care activities in this specific study. Patients treated in surgical units are more satisfied with the nursing care they receive according to Geçkil et al. (2008). Nursing care needs to be taught and delivered in a professional manner value to be effective and valuable both for patients and caring nurses. Attention is given to a person, and the importance of patient‐focused care in improving nursing care behaviours is important which should be taken into consideration.

However, nursing care is not limited to only its’ technical aspects; it is a profession that requires plenty of both technical and psychological skills which includes knowing and treating the patient as an individual. Some studies point out that nursing students usually choose nursing as a career choice because of easy employment status rather than their capability to do it (Cho et al., 2010). In Turkey, university students usually choose their careers according to their grades after university entrance examinations. So, there is no way to determine the ability of students who enter into nursing schools in current practice. Choosing a profession only because of easy employment status also could be a reason for different perceptions about nursing care besides difficulties raise by working conditions itself.

### 5.3 Limitations

One of the limitations of this study was nurses who were employed in one university hospital were included. Although the results of this study cannot be generalized to all the nurses in Turkey, findings of this study are quite similar to the ones done previously with nursing students. Another limitation of the study was that there are limited studies related to perceptions of nurses about caring in Turkey.

## 6 CONCLUSION

In conclusion, the results of this specific study suggest that technical aspects of nursing were mostly endorsed items in CDI‐25 except for the item providing privacy for the patient. It has been perceived that although some cultural factors do influence perceptions of nurses about caring, they usually focus on the technical skills of nurses. The influence of therapeutic communication skills, the success of the nurse–patient communications and the cultural values of nurses may change nurses’ perception of nursing care. Different studies related to caring in nursing should be conducted to specify why a psychosocial aspect of nursing is not considered a priority by nursing staff. Also, studies related to caring in nursing should be conducted to specify how cultural factors influence the perceptions of caring among nurses.

## 7 CONTRIBUTIONS

NA, RW, NV and AÖ: Study Design. NA and AÖ: Data collection. RW: Data analysis and Review of the manuscript. NA, RW, NV and AÖ: Manuscript preparation.

All authors approved the final version of the manuscript.

## CONFLICTS OF INTEREST

The authors declare that there are no potential conflicts of interest related to research, authorship and publishing of this article.

## Data Availability

Data can be provided by the corresponding author on reasonable request.
